# Fabrication of PLA-Based Electrospun Nanofibers Reinforced with ZnO Nanoparticles and *In Vitro* Degradation Study

**DOI:** 10.3390/nano13152236

**Published:** 2023-08-02

**Authors:** Valentina Salaris, Iñaki San Félix García-Obregón, Daniel López, Laura Peponi

**Affiliations:** Instituto de Ciencia y Tecnología de Polímeros (ICTP-CSIC), C/Juan de la Cierva 3, 28006 Madrid, Spain; v.salaris@ictp.csic.es (V.S.); i.sanfelixupm@outlook.com (I.S.F.G.-O.)

**Keywords:** electrospinning, PLA, ZnO nanoparticles, in vitro degradation, woven no-woven mats, mechanical properties

## Abstract

In this work, electrospun nanofibers based on polylactic acid, PLA, reinforced with ZnO nanoparticles have been studied, considering the growing importance of electrospun mats based on biopolymers for their applications in different fields. Specifically, electrospun nanofibers based on PLA have been prepared by adding ZnO nanoparticles at different concentrations, such as 0.5, 1, 3, 5, 10 and 20 wt%, with respect to the polymer matrix. The materials have been characterized in terms of their morphological, mechanical, and thermal properties, finding 3 wt% as the best concentration to produce PLA nanofibers reinforced with ZnO nanoparticles. In addition, hydrolytic degradation in phosphate buffer solution (PBS) was carried out to study the effect of ZnO nanoparticles on the degradation behavior of PLA-based electrospun nanofiber mats, obtaining an acceleration in the degradation of the PLA electrospun mat.

## 1. Introduction

The solution electrospinning technique is a processing method that permits the fabrication of polymeric fibers by applying a high voltage between a spinneret and a conductive collector [1]. As the intensity of the electrical field is increased, the pendant drop of the polymeric solution is deformed into a conical shape called a Taylor cone. When a critical voltage is reached, the charged jet is ejected and stretched into random fibers, in the range from micro to nano, which subsequently undergoes whipping motions [2]. Furthermore, the quick evaporation of the solvents leads to fast solidification of the polymer, which is collected as woven, no-woven fibers mats on the collector [3]. The fibers diameter can be tailored by optimizing various parameters related to the polymeric solution and processing conditions. Environmental parameters such as temperature and humidity also affect the morphology [4]. In particular, electrospun micro and nanofibers have gained attention for their use in various applications such as biological engineering, wound dressing, drug delivery, energy storage, sensors, packaging, etc. [5,6,7,8].

All soluble and fusible melt polymers are appropriate for the electrospinning technique, performing solution or melt electrospinning, respectively. Moreover, both natural and synthetic polymers can be used as well as biodegradable and biocompatible polymers represent an excellent alternative to polymers derived from fossil fuels. Among these, poly(lactic acid) (PLA) is one of the most used [9]. PLA is very attractive for its biodegradability and biocompatibility, making it an option for fabricating tissue engineering materials such as scaffolds, implantable devices and drug delivery systems [10,11,12]. Furthermore, PLA presents relatively high modulus and strength but poor toughness, low degradation rate, and high hydrophobicity [13,14].

To improve mechanical, chemical and physical properties, researchers use one of the main approaches to developing nanocomposites constituted by a polymeric matrix reinforced with nanoparticles (NPs), which can be either organic or inorganic [15].

The effect of the introduction of NPs in PLA-based electrospun nanofibers has already been studied. Leones et al. [16] studied PLA’s thermal and mechanical properties and shape memory behavior reinforced with magnesium oxide (MgO) NPs. Tazibt et al. [17] evaluated the effect of hydroxyapatite (HA) on the morphological and physical properties of PLA-HA composites. Among inorganic NPs, zinc oxide (ZnO) has gained interest in the last years since it is recognized as bio-safe material. It is used to produce cosmetics by US Food and Drug Administration [18], apart from its applications in numerous sectors such as food packaging, tissue engineering, cosmetics, membranes, pharmaceutical products and electronic devices [19,20,21,22].

Additionally, ZnO NPs have been widely used because of their antibacterial, optical, piezoelectric and mechanical properties [23]. It is reported that incorporating this type of NPs can improve the mechanical properties of resistance to fatigue and toughness, besides nanocomposites’ optical and barrier properties [24]. At the same time, ZnO NPs have already been incorporated into PLA to study the produced materials’ antibacterial activity and mechanical and physicochemical properties [25,26,27].

Furthermore, PLA is widely used for biomedical applications, particularly tissue engineering applications, as absorbable implants, sutures and other devices [10]. The degradation of PLA in physiological conditions occurs through hydrolysis cleavage of the ester bond, leading to the formation of shorter polymeric fragments, which are soluble in water and quickly metabolized by the human body [28]. However, its degradation rate can vary between 2 and 5 years depending on its molecular weight and degree of crystallinity since degradation involves first the amorphous region of the polymer [29].

The incorporation of NPs is proven to increase the degradation rate of polymers like PLA by increasing the hydrophilicity of the material and therefore increasing the amount of absorbed water [30].

In the present work, PLA-based electrospun nanofibers reinforced with ZnO NPs at different concentrations, 0.5, 1, 3, 5, 10 and 20 wt% with respect to PLA are described in terms of morphological, mechanical, and thermal properties. Furthermore, considering the potential application of these materials in the biomedical field, a detailed study of the degradation in simulated physiological conditions and the effect of ZnO NPs in the degradation behavior of the fabricated electrospun nanofibers mats was carried out. In particular, phosphate buffer saline (PBS), a buffer solution with pH ≈ 7.4, was chosen as a biological medium for preliminary in vitro degradation studies.

## 2. Materials and Methods

### 2.1. Materials

Polylactic acid (PLA 3052D) 3% of D-lactic acid monomer, molecular weight 142,000 g/mol, density 1.24 g/cm) was kindly supplied by NatureWorks^®^, Minneapolis, MN, USA. Zinc oxide nanoparticles (ZnO NPs, purity: 99.5%, APS: 10–30 nm) were purchased from Nanoshel^®^, Amritsar, India. Chloroform (CHCl_3_) (99.6% purity) and N, N-dimethylformamide (DMF) (99.5% purity) from Sigma Aldrich (St. Louis, MO, USA) were used as solvents.

PLA and PLA-based nanofibers were prepared by adding different amounts of ZnO NPs, that is 0.5, 1, 3, 5, 10 and 20 wt%, with respect to the polymeric matrix. Briefly, ZnO NPs were dispersed with a sonicator tip (Sonics Vibra-Cell VCX 750, Fisher Scientific, Hampton, NH, USA) for 1 h and 30 min. Afterwards, PLA solution (10 wt% in chloroform) stirred overnight was added and dispersed for 1 h. Finally, DMF was added to obtain a solvent mixture of CHCl_3_:DMF (4:1).

Electrospun mats were obtained in an Electrospinner Y-flow 2.2D-XXX (Y-Flow, Malaga, Spain) by applying a voltage of 20 kV, a flow rate of 2 mL/h and 0.5 mL/h for the suspension and the solvent mixture, respectively, and with a distance needle-collector of 14 cm. The electrospun nanofibers mats were electrospun for 3 h and dried for 24 h in a vacuum chamber before the characterization.

### 2.2. Characterization Techniques

Scanning Electron Microscopy, SEM (PHILIPS XL30 Scanning Electron Microscope, FEI Philips, Hillsboro, OR, USA), was used to study the morphology of the electrospun nanofibers and the effect of the degradation in the nanofibers. All the samples were previously gold-coated (≈5 nm thickness) in a Polaron SC7640 Auto/Manual Sputter. SEM images were acquired at different magnitudes, in particular, 1500×, 4000×, and 8000× with an accelerating voltage of 25.0 kV and a working distance of 15 mm. SEM images were analyzed with ImageJ software, and the porosity and diameters were calculated as the average value of 30 random measurements for each sample.

The presence and the distribution of ZnO NPs were studied by field emission scanning electron microscopy, FESEM (Hitachi S8000, Hitachi Co., Tokyo, Japan), using an accelerating voltage of 30 kV and a working distance of 8.3 mm.

X-ray diffraction measurements (XRD) were carried out using a Bruker D8 Advance instrument with a CuK as source (0.154 nm) and a Detector Vantec1. The scanning range was 2° and 80°, and the step size and count time per step were 0.023851° and 0.5 s.

Attenuated total reflectance-Fourier transform infrared spectroscopy (ATR-FTIR) measurements were performed by a Spectrum One FTIR spectrometer (Perkin Elmer instruments) in the range of 4000–400 cm^−1^ with a resolution of 4 cm^−1^ and acquiring four scans for each spectrum. The spectra were processed using OriginPro 8.5.

Thermogravimetric analysis (TGA) was performed in a TA Instrument, TGA Q500 thermal analyzer. The experiments were carried out under a nitrogen atmosphere (flow rate of 60 mL/min). Samples were heated from room temperature to 800 °C with a heating rate of 10 °C/min. The maximum degradation temperature (T_max_) was calculated from the first derivative of the TGA curves.

Thermal transitions were analyzed by Differential Scanning Calorimetry in a DSC Q2000 TA Instruments under a nitrogen atmosphere (50 mL/min). The thermal analysis was programmed at 10 °C/min from −10 to 200 °C, obtaining the glass transition temperature (T_g_), calculated as the midpoint of the transition, the cold crystallization temperature (T_cc_) and the melting temperature (T_m_). The degree of crystallinity (Xc%) was calculated using Equation (1).

(1) $$Xc\%=100\times \frac{\Delta H_{m}-\Delta H_{cc}}{\Delta H_{m}^{{}^{\circ}}}$$

where ΔH_cc_ and ΔH_m_ represent the cold crystallization and the melting enthalpy, respectively. Taking the value of crystallization enthalpy of pure crystalline PLA, $\Delta H_{m}^{{}^{\circ}}$ as 93.6 J/g [16].

Tensile tests were carried out with a QTest 1/L Elite instrument using a 100 N load cell and applying a strain rate of 10 mm/min and an initial length of 20 mm.

The values of elastic modulus, tensile stress and elongation at break were taken by five specimens of 20 mm length, 5 mm width and 30–50 µm of average thickness.

One-way analysis of variance (ANOVA) was performed with the program Statgraphics Centurion XVII (Statpoint Technologies, Inc., Warrenton, VA, USA). To study the groups significantly different from others, Tukey’s test was carried out with a 95% confidence level.

The in vitro degradation study was carried out by immersing samples of 1 cm^2^ of each PLA-based electrospun fiber mat in 20 mL of phosphate-buffered saline (PBS) and maintained at 37 °C in an oven throughout the experiment. The PBS medium was changed each week, and the pH was measured with a pH METER-02 (Homtiky) with an error of ±0.01. The degradation process was studied at different times, named TX, where x indicates the immersion time, at 1, 3, 7, 14, 21, 28, 84 until 240 days, which was taken as the last day of the experiment, and compared to T0, which was considered as reference. The samples were dried for 14 days in a vacuum chamber and characterized with the above characterization techniques.

## 3. Results

The morphology of electrospun PLA (ePLA) and PLA-based nanofibers mats have been characterized by SEM analysis. All the nanofibers presented an average diameter of a few hundred nanometers.

**Figure 1:**
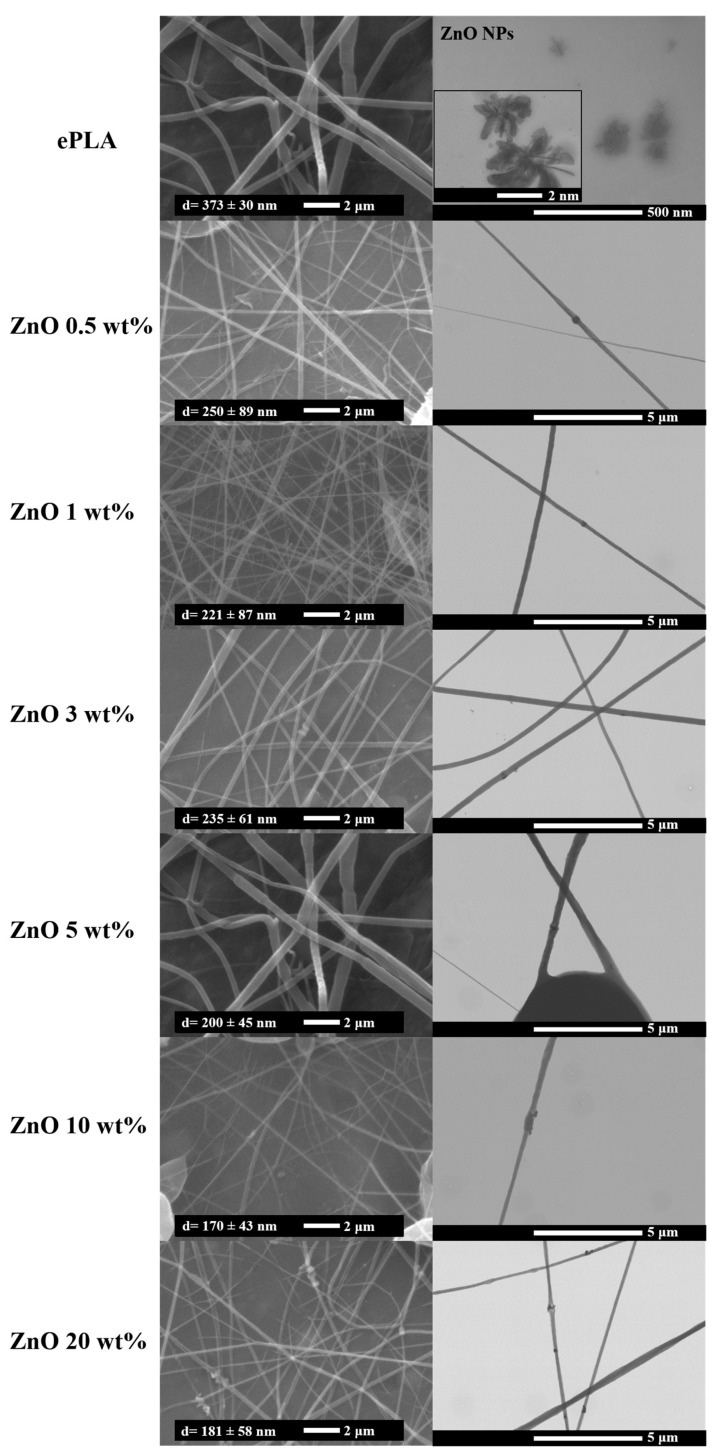
SEM and FE-SEM images of ePLA and PLA-based nanofibers.

Figure 1 are reported the fabricated electrospun nanofibers with their average diameter and FE-SEM analysis, where it is possible to see the presence of NPs inside the fibers demonstrating the good incorporation of ZnO NPs into the electrospun fibers. Randomly-oriented e-fibers were obtained, and ePLA presented a diameter distribution of 373 ± 30 nm, according to values already reported in the literature [31]. However, it was observed that the main diameter of the electrospun fibers decreased by increasing the NPs concentration.

The diameter decreased from 373 ± 30 nm for neat ePLA to 170 ± 43 nm and 181 ± 58 nm with 10 wt% and 20 wt% of NPs, respectively, with a reduction in the average diameter of almost 54% and 51%. This phenomenon may be due to an increase in conductivity and a consequent decrease in viscosity, both caused by the presence of ZnO NPs leading to a reduction in diameter [32]. Goncharova et al. [26] fabricated poly-L-lactic acid (PLLA)/ZnO composite membranes and observed a decrease in fiber diameter of almost 40% with the incorporation of ZnO NPs in all percentages.

This phenomenon was also observed in other systems with other NPs, such as magnesium oxide (MgO) and cellulose nanocrystals (CNC), which was attributed to the increased conductivity of the solution due to the presence of nanoreinforcements [16,33].

**Figure 2:**
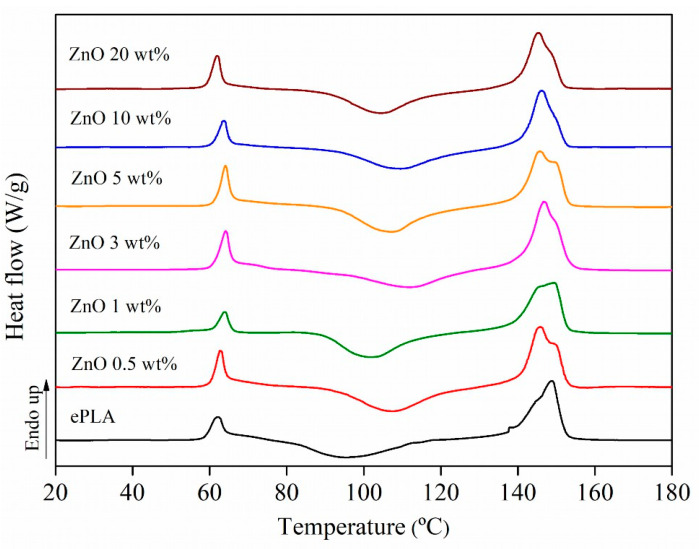
DSC analysis of ePLA and PLA-based nanofibers.

DSC analysis has been carried out to study the thermal behavior of ePLA and PLA-based nanofibers. The thermograms are reported in Figure 2, and the results are in Table 1.

All the materials presented a glass transition temperature (T_g_) around 60 °C. Thus the presence of NPs does not significantly affect the T_g_ of PLA. The cold crystallization temperature (T_cc_) increased with all concentrations of NPs of almost 10 °C, from 96 °C for neat ePLA to above 100 °C for all nano-reinforced nanofibers. Moreover, ePLA showed a melting temperature (T_m_) of around 146 °C. However, by adding ZnO nanoparticles, the presence of two melting peaks around 145 °C and 149 °C has been observed, maybe related to the formation of crystals of different sizes [34].

ZnO NPs can increase the crystallinity of neat polymeric matrix. Indeed X_c_ increased from 3% for ePLA reaching the highest value of 10% with PLA + ZnO 3 wt%. The increase in crystallinity can be attributed to the fact that NPs are acting as nucleating agents and promoting the nucleation of the polymeric chains [35], while at higher concentrations, above 5 wt%, this increment was followed by a decrease probably caused by the presence of small NPs aggregations that lower the chains mobility leading to the production of less crystalline materials [36].

**Figure 3:**
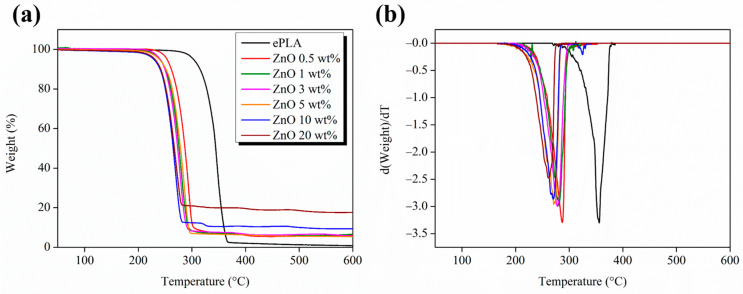
Thermogravimetric analysis of ePLA and PLA-based electrospun nanofibers (**a**) weight vs. temperature and (**b**) derivative curves.

Furthermore, TGA analysis has been carried out to study our materials’ thermal stability, and the results are reported in Figure 3.

All nanofibers showed a single degradation step, except nanofibers with 10 wt% of ZnO NPs, which showed two degradation steps. In particular, neat ePLA presented the highest T_max_ of 355 °C, which decreased by almost 95 °C until 260 °C by adding 20 wt% of ZnO NPs. ZnO NPs decreased the thermal stability of PLA nano-reinforced nanofibers, probably due to the degrading effect of ZnO, as reported by Shankar et al. [37].

Indeed, the presence of ZnO NPs can catalyze either a transesterification reaction or an “unzipping” depolymerization through the coordination of ZnO with the carbonyl groups of the polymeric chains, leading to a decrease in the thermal stability of the materials [38].

**Figure 4:**
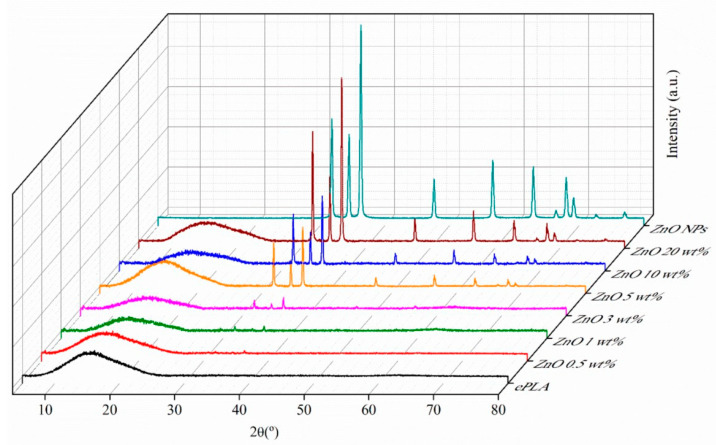
XRD analysis of ePLA and PLA-based electrospun nanofibers.

Moreover, XRD analysis has been carried out to confirm further the presence of NPs in the nanofibers. In Figure 4, XRD patterns of ePLA and PLA-based nanofibers are reported.

ePLA showed a broad peak at 2θ = 15° demonstrating the amorphous nature of ePLA [31], due to the fast evaporation of the solvent, avoiding the mobility of the polymeric chains and leading to the production of a material with a very low crystallinity degree during the formation of fibers in the electrospinning process. Furthermore, the diffraction pattern of ZnO at 2θ = 31.8°, 34.5°, 36.3°, 47.6°, 56.6° and 62.9° of the lattice planes (100), (002), (101), (102), (110) and (103), respectively can be observed [39]. The intensity of the signals increased by increasing the NPs content proving the good incorporation of ZnO NPs.

Mechanical characterization was carried out by tensile test measurement. In Table 2 are reported the values of elastic Modulus (E), tensile strength (σ), and elongation at break (ε at break). Neat ePLA showed an E value of 60 ± 5 MPa and σ of 5.1 ± 0.5 MPa. However, incorporating ZnO NPs leads to a significant reduction of both E and σ in all concentrations. It was observed a decrease from 60 ± 5 MPa to 11.6 ± 2.9 and to 5.5 ± 1.9 for ZnO 0.5 and 1 wt%, respectively, while a further increment was observed with 3 wt% reaching a value of 21.5 ± 4 MPa.

The mechanical response finally drops down by adding 5, 10, and 20 wt% of ZnO, probably caused by small NPs agglomeration presented in the nanocomposite [26,40].

**Figure 5:**
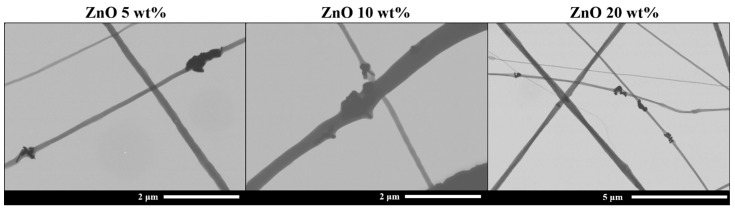
FE-SEM images of 5, 10 and 20 wt% samples with NPs agglomerations.

Figure 5 shows FESEM images of 5, 10 and 20 wt% electrospun mats where small agglomerates can be observed. These agglomerates, of about 0.6–1 µm in size, can act as “defects”, reducing the stress transfer ability from the electrospun nanofibers to the reinforcements.

The same behavior was observed with σ. Other researchers reported a decrease in tensile strength by adding ZnO NPs. For example, Murariu et al. [38] recorded a reduction in σ of ~50% by adding 2 and 3% of ZnO to a PLA matrix.

It is important to remark that although ZnO NPs caused a decrease in E and σ in all concentrations, ePLA + ZnO 3 wt% showed the best mechanical properties among the PLA-based nanofibers mats produced in this work. The material with 3 wt% of reinforcements presented the highest crystallinity. The ε at the break as well decreased in all concentrations of NPs, except for PLA + ZnO 3 wt%, which presented an ε at break of 38.8 ± 5.6 similar to ePLA (45.7 ± 10). This non-linear behavior of the mechanical properties concerning the different numbers of nanoparticles added to the polymeric matrix has also been confirmed by ANOVA analysis. The sample with 3 wt% ZnO NPs shows a significance different to the other reinforced samples for each mechanical property studied.

Moreover, in the case of the elongation at break, its value is significant to the values obtained for neat ePLA mat. Therefore, samples reinforced with 0.5 and 1 wt% concentrations show significance among themselves and those with the highest concentrations, such as 5, 10 and 20 wt%. In the first case, the number of NPs is low to promote a matrix-reinforcement effect, while 5, 10, 20 wt% are high concentrations, and the NPs tend to aggregate, as shown in Figure 5.

The porosity of the mats also affects the mechanical response of the materials [41]. Therefore, the decrease in the mechanical performances could also be related to the increase in porosity observed in the reinforced nanofibers mats with respect to the neat polymeric matrix. Furthermore, the porosity of the fibers was calculated. ePLA presented a porosity of 18%, while PLA-based nanofibers showed higher porosity (above 27%), which could be attributed to the reduction in diameter caused by the incorporation of NPs.

Canales et al. [42] also reported a decrease in both E and σ by adding 10 and 20 wt% of ZnO in PLA nanofibers. Furthermore, the decrease of mechanical properties caused by incorporating ZnO NPs was also observed with other polymeric matrices such as PCL. Shitole et al. [43] obtained a concentration-dependent reduction in tensile strength by incorporating 1, 5, 10, 15, and 30 wt% of ZnO NPs into PCL/nHA matrix.

Even if nano-reinforced nanofibers did not improve mechanical response, the values of E and σ are quite similar to those of human tissues, such as human skin and aortic valve [16].

**Figure 6:**
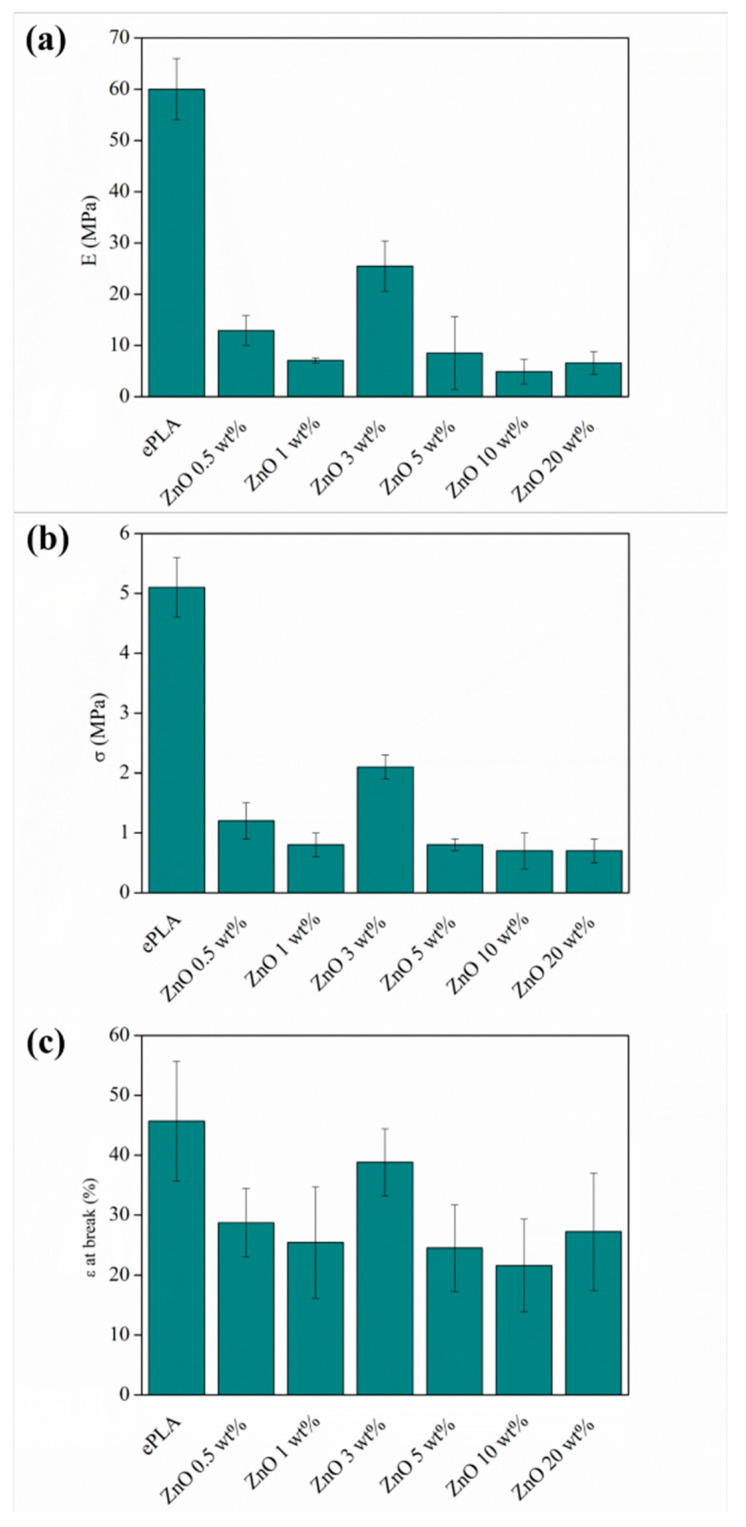
Schematic representation of the variation of (**a**) E, (**b**) σ, and (**c**) ε at break of ePLA and PLA-based nanofibers.

Figure 6 are summarized the obtained results of E, σ, and ε at the break to better visualize the variation of these three parameters with respect to the different amounts of NPs added to the ePLA.

Furthermore, the in vitro degradation study in PBS was carried out to evaluate the degradation behavior of ePLA and the effect of ZnO NPs on the degradation of reinforced nanofibers.

**Figure 7:**
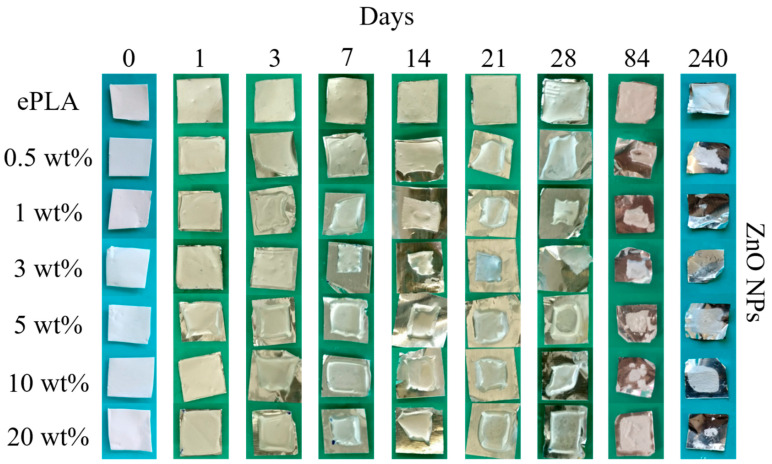
The visual appearance of ePLA and PLA-based electrospun nanofibers at different immersion times.

Figure 7 shows the visual appearance of the mats of ePLA and nanofibers with ZnO NPs after 1, 3, 7, 14, 21, 28, 84, and 240 days, respectively.

It can be observed that neat ePLA does not present particular differences even after 240 days, while PLA-based nanofibers in all concentrations showed contraction of the mat already at T1, probably due to the hydrophilic properties of ZnO NPs [19] and visual degradation of the mat already at T84.

From the macroscopical point of view, reinforced nanofibers seem to show faster degradation with respect to neat ePLA.

**Figure 8:**
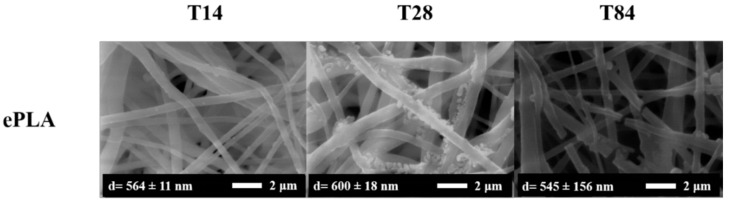
SEM analysis of ePLA at T14, T28 and T84.

The morphology of the degraded fibers was studied by SEM. In Figure 8 are reported SEM images of ePLA at T14, T28, and T84 with their average diameter.

No particular changes were observed at T14, while a mineralization process in the nanofibers surface can be observed after 28 days, and the rupture of fibers after 84 days was caused by the degradation.

It is essential to point out that an increase in diameter was observed during the degradation test from the initial value of 373 nm to 600 nm after 28 days to a subsequent decrease until 545 nm after 84 days, however, with a broad diameter distribution.

**Figure 9:**
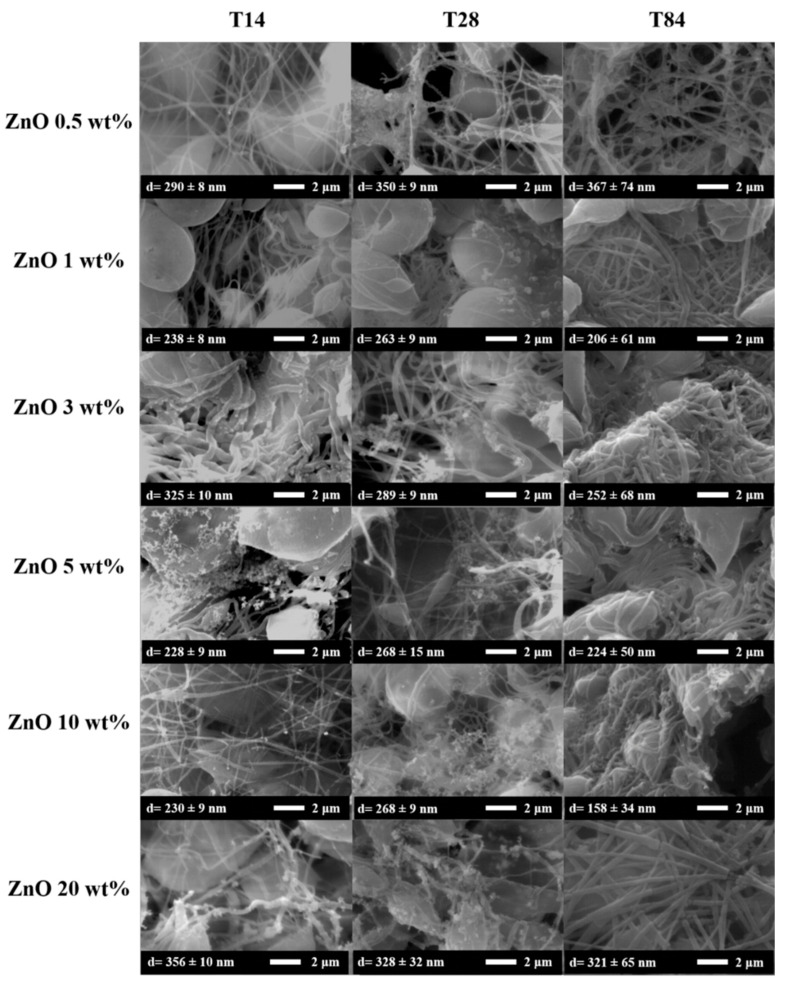
SEM images of PLA-based nanofibers at T14, T28 and T84, respectively.

Figure 9 reported the SEM images of ePLA-based nanofibers with different percentages of NPs and at other immersion times. The nanofibers lost their morphology after 14 days, and the precipitation of salts from the solution was also observed in this case, confirmed by XRD analysis. Furthermore, broken fibers can be easily observed, particularly with 20 wt% of ZnO at T84.

In this case, the nanofibers also swell during the degradation process. The diffusion of water is easier, both because of the hydrophilicity of ZnO NPs [19] and because of the low crystallinity of our materials. For this reason, amorphous polymers tend to undergo bulk erosion instead of surface erosion, which is typical of crystalline polymers such as polycaprolactone (PCL) [44].

**Figure 10:**
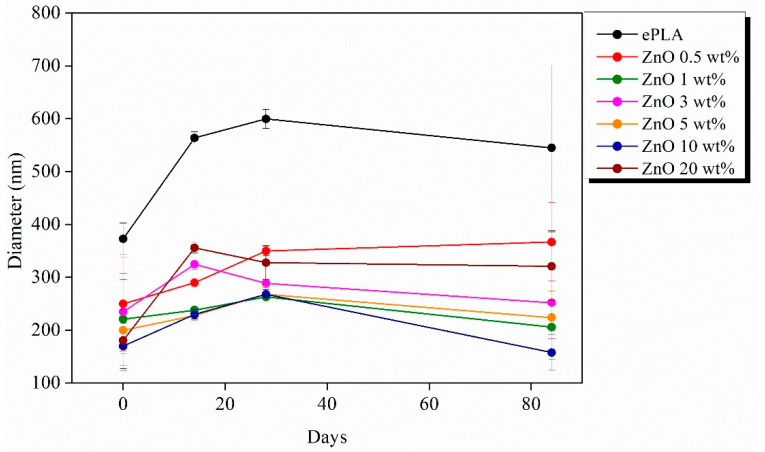
Variation of the average diameters of electrospun nanofibers during the degradation test.

Figure 10 is reported the variation of the average diameter at different immersion days. All the electrospun nanofibers showed an increase after 14 days. In particular, neat ePLA presented an increase of almost 50% at T14 and almost 60% after 28 days. However, the average diameter further decreased after 84 days without reaching the initial value.

Nanofibers reinforced with ZnO NPs showed the same behavior. In the case of ZnO 0.5, 1, 5 and 10 wt%, the average diameter increased until T28 and then decreased at T84, while nanofibers reinforced with 3 and 20 wt% of NPs showed an increase until T14 followed by a decrease until T84.

Furthermore, to better study the degradation process of electrospun nanofibers, the materials were characterized through FT-IR analysis.

**Figure 11:**
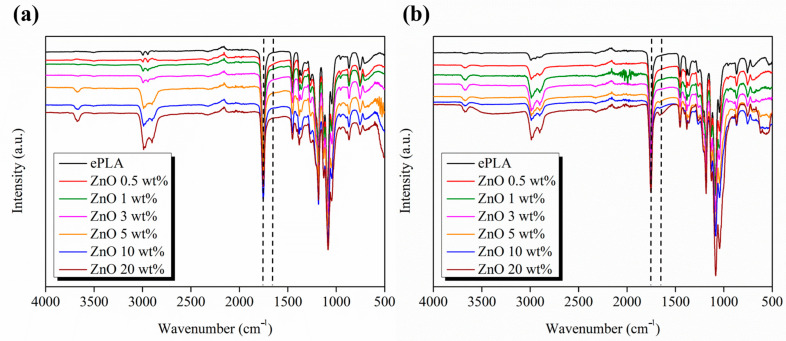
FT-IR spectra of the fabricated materials at (**a**) T0 and (**b**) T84.

In Figure 11, the FT-IR spectra recorded in the range 4000–400 cm^−1^ of each sample at T0 are compared to the spectra at T84. The signals at 2981 cm^−1^ and 2898 cm^−1^ can be attributed to the asymmetrical and symmetrical stretching vibrations of the CH_2_ bond. The sharp peak at 1747 cm^−1^ is assigned to the stretching of the carbonyl group, while the signal at 1455 cm^−1^ and 1370 cm^−1^ to the CH_3_ deformation vibration.

Furthermore, the signals at 1182 cm^−1^ and 1085 cm^−1^ correspond to the binding vibrations of the C-O-C ether bond [45]. No particular differences were observed between the PLA spectrum and reinforced nanofibers.

Hydrolytic degradation of PLA occurs through cleavage of the ester bond. Hydrolysis leads to carboxylate and hydroxyl groups forming, which present characteristic peaks at 1650 cm^−1^ and a broad peak at 3400 cm^−1^, respectively [46].

The degrading effect of ZnO NPs was studied also with other polymeric matrices, such as PCL, Augustine et al. [47] studied the in vitro degradation of PCL-based electrospun nanofibers in SBF also by FT-IR, observing a higher degradation rate of PCL/ZnO composites compared to neat PCL.

Abdalkarim et al. [48] observed that incorporating ZnO into the PHBV matrix increased the hydrophilicity of the nanocomposite and then its degradation rate.

**Figure 12:**
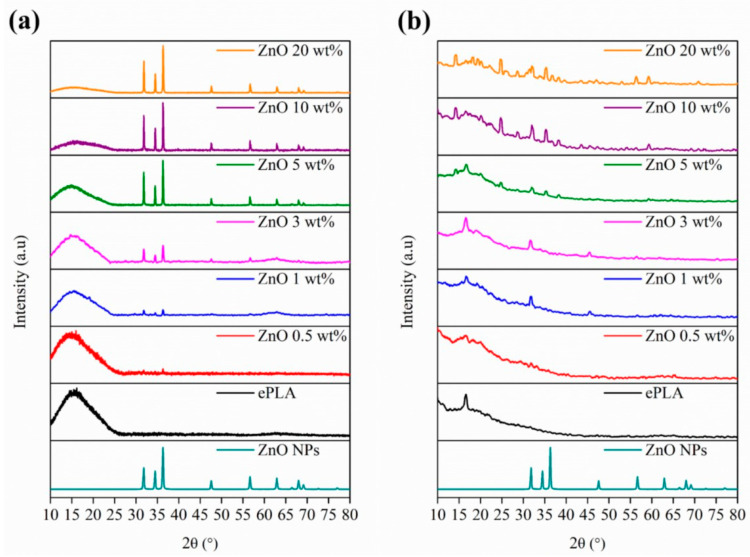
XRD pattern of electrospun nanofibers at (**a**) T0 and (**b**) T84.

The materials were also characterized by XRD analysis, and the diffraction patterns of ePLA and PLA-electrospun-based nanofibers at T0 and after 84 days of degradation are shown in Figure 12. After 84 days, new peaks appear at 2θ = 32.1°, 45.7°, and 56.5° that can be attributed to the crystallographic planes of NaCl [49] due to the precipitation of salts.

Other signals at 2θ = 14.4°, 16.7°, 18.2°, 20.1°and 28.8° can be associated with the α-form of PLA, which present a 10_3_ helical chain conformation [50].

Indeed, chain cleavage reaction during the hydrolytic degradation of PLA occurs first preferentially in amorphous regions leading to an increase in polymer crystallinity this reason, at T0, PLA presented a typical amorphous pattern while at T84, all the [51]. For materials show new signals related to the crystalline form of PLA.

**Figure 13:**
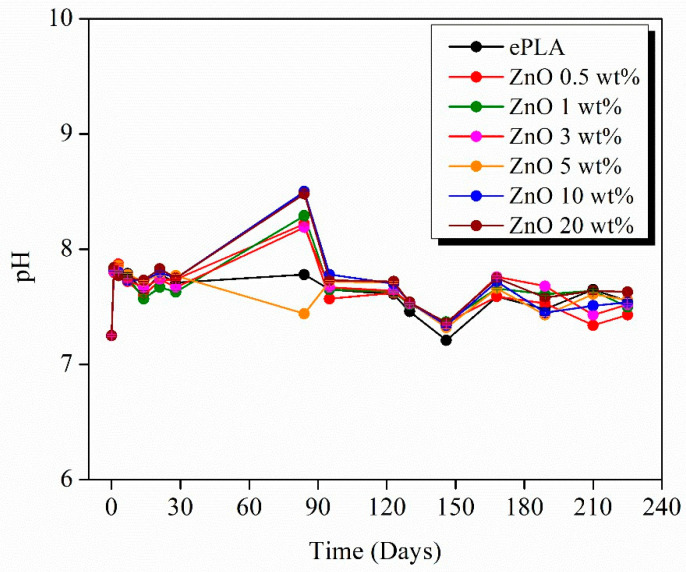
pH evolution during the degradation test.

Since the degradation of PLA leads to the formation of acidic products, it is important to study the pH evolution during the entire process (Figure 13). pH was measured every seven days in the first weeks and then every two weeks when no significant changes were observed. The pH measurements were carried out at room temperature.

The pH value of around 7.5 was retained during the degradation. However, a rise was observed after 84 days until values above 8, which can be attributed to the release of Zn ions [44], except for neat PLA and ZnO 5 wt%, which decreased pH.

The reduction in diameter at T84 was observed after the swelling of nanofibers for the first 28 days. Furthermore, all materials, except ePLA, showed a contraction of the mat until T28 and an essential loss of the material directly at T84.

Therefore, T84 could coincide with the maximum release of Zn ions, which leads to the formation of Zn(OH)_2_ and, consequently, an increase in pH [52].

The same behavior was noticed with other types of reinforcement. For example, Leones et al. [53] fabricated PLA-Mg filaments for 3D printing applications, observing that 100% of Mg was already released after 84 days when 15 wt% Mg was added.

All the materials presented a decrease after 146 days until values around 7.20 to further increase until 7.5 and maintaining constant until the end of the degradation test.

## 4. Conclusions

ePLA and PLA-based randomly orientated nanofibers were successfully produced at different NPs concentrations.

The good incorporation of ZnO NPs was confirmed by FE-SEM and XRD analysis by observing the characteristic pattern of ZnO NPs, whose signals increased by increasing the NPs concentration. It was observed that the presence of ZnO NPs reduces the average diameter of neat PLA by about 54% and 51%, with 10 and 20 wt% of NPs, respectively, probably caused by an increase in the conductivity of the solution. Furthermore, NPs decrease the thermal stability and mechanical performance of nanofibers. Indeed PLA showed the highest E and σ values. However, the incorporation of ZnO NPs significantly decreased both values. Among the reinforced nanofibers produced in this work, electrospun nanofibers with 3 wt% of NPs showed the best mechanical properties, which is also the sample that presented the highest crystallinity, while a reduction was observed with higher concentrations, probably related to the presence of NPs agglomerations that could be connected to the reduction in mechanical properties.

Moreover, the hydrolytic degradation of the materials was studied in PBS at different immersion times for almost one year. In particular, ePLA presented similar properties and the same visual appearance even after 240 days, while ePLA-based nanofibers show changes in terms of contraction and degradation of the materials after 84 days.

From SEM analysis, it was observed that nanofibers lost their morphology, and the breakage of fibers was observed at T84 with 20 wt% of ZnO. The degradation of the fabricated materials was also confirmed by FT-IR and XRD analysis. After 84 days, the diffraction pattern of ZnO NPs disappeared while new peaks corresponding to α-form of PLA confirmed the increased crystallinity of the materials since the degradation involves first the amorphous regions. Furthermore, the pH is maintained around the physiological value during the entire degradation process.

Thus, considering all the results obtained, we can conclude that ZnO NPs can accelerate the degradation process of a polymeric matrix as PLA, reducing its degradation time.

## Figures and Tables

**Table 1:** DSC characterization of electrospun mats.

	T_g_ (°C)	T_cc_ (°C)	T_m_ (°C)	X_c_ (%)
ePLA	60	96	149	3
ePLA + ZnO 0.5 wt%	61	107	145/149	5
ePLA + ZnO 1 wt%	61	102	146/149	4
ePLA + ZnO 3 wt%	62	112	146	10
ePLA + ZnO 5 wt%	62	107	146/149	2
ePLA + ZnO 10 wt%	61	109	146	3
ePLA + ZnO 20 wt%	59	105	145	4

**Table 2:** Mechanical characterization of electrospun nanofibers mats.

Sample	E (MPa)	σ (MPa)	ε at Break (%)	Porosity %
ePLA	60 ± 5 ^a^	5.1 ± 0.5 ^a^	45.7 ± 10 ^a^	18
ePLA + ZnO 0.5 wt%	11.6 ± 2.9 ^c^	1.2 ± 0.3 ^b^	28.7 ± 5.7 ^b^	30
ePLA + ZnO 1 wt%	5.5 ± 1.9 ^c,d^	0.8 ± 0.2 ^b^	25.4 ± 9.3 ^b^	38
ePLA + ZnO 3 wt%	21.5 ± 4 ^b^	2.1 ± 0.2 ^c^	38.8 ± 5.6 ^a^	29
ePLA + ZnO 5 wt%	7.2 ± 3.8 ^c,d^	0.8 ± 0.1 ^b^	24.5 ± 7.2 ^b^	27
ePLA + ZnO 10 wt%	4.9 ± 2 ^d^	0.7 ± 0.3 ^b^	21.6 ± 7.7 ^b^	33
ePLA + ZnO 20 wt%	4.2 ± 1.4 ^d^	0.7 ± 0.2 ^b^	27.2 ± 9.8 ^b^	37
F ratio	191.19	125.48	8.35	
*p*-Value	0.0000 *	0.0000 *	0.0000 *	

Different letters in the column indicate significant differences according to Tukey’s test (*p* < 0.05). * Values are significant at *p* < 0.05.

## Data Availability

The data presented in this study are available on request from the corresponding author.
